# *NtRNF217*, Encoding a Putative RBR E3 Ligase Protein of *Nicotiana tabacum*, Plays an Important Role in the Regulation of Resistance to *Ralstonia solanacearum* Infection

**DOI:** 10.3390/ijms22115507

**Published:** 2021-05-24

**Authors:** Ying Liu, Yuanman Tang, Xi Tan, Wei Ding

**Affiliations:** College of Plant Protection, Southwest University, Chongqing 400715, China; yingliu2020@swu.edu.cn (Y.L.); tangym08@163.com (Y.T.); tanxi2020@email.swu.edu.cn (X.T.)

**Keywords:** *NtRNF217*, RBR E3 Ligase, *Nicotiana tabacum*, overexpression experiments, *Ralstonia solanacearum*

## Abstract

E3 ubiquitin ligases, the most important part of the ubiquitination process, participate in various processes of plant immune response. RBR E3 ligase is one of the E3 family members, but its functions in plant immunity are still little known. NtRNF217 is a RBR E3 ligase in tobacco based on the sequence analysis. To assess roles of *NtRNF217* in tobacco responding to *Ralstonia solanacearum*, overexpression experiments in *Nicotiana tabacum* (Yunyan 87, a susceptible cultivar) were performed. The results illuminated that *NtRNF217*-overexpressed tobacco significantly reduced multiplication of *R. solanacearum* and inhibited the development of disease symptoms compared with wild-type plants. The accumulation of H_2_O_2_ and O_2_^−^ in *NtRNF217*-OE plants was significantly higher than that in WT-Yunyan87 plants after pathogen inoculation. The activities of CAT and SOD also increased rapidly in a short time after *R. solanacearum* inoculation in *NtRNF217*-OE plants. What is more, overexpression of *NtRNF217* enhanced the transcript levels of defense-related marker genes, such as *NtEFE26*, *NtACC Oxidase*, *NtHIN1*, *NtHSR201*, and *NtSOD1* in *NtRNF217*-OE plants after *R. solanacearum* inoculation. The results suggested that *NtRNF217* played an important role in regulating the expression of defense-related genes and the antioxidant enzymes, which resulted in resistance to *R. solanacearum* infection.

## 1. Introduction

The ubiquitin-proteasome system (UPS) is widely found in eukaryotes and plays an important role in post-translational modification of proteins in cells [1]. This system is involved in regulation of biological growth and development, as well as the adaptability of organisms to the surrounding environment [2]. The ubiquitin modification of the target protein requires the sequential action of three enzymes: E1 ubiquitin-activating enzymes, E2 ubiquitin-conjugating enzymes, and E3 ubiquitin ligases [3]. In plants, there are a large number of E3 ubiquitin ligase gene families. For example, *Arabidopsis* has more than one thousand E3 ubiquitin ligase genes based on the genome analysis, while there are only a small number of E1 and E2 genes [1,4,5].

According to the structure and mechanism of action, E3 ubiquitin ligases can be divided into different categories including RING (Really Interesting New Gene), U-box, HECT (homologous to E6-associated protein C-terminus), and RBR (RING1-IBR-RING2) [5,6]. The RING and U-box type ligases can function as molecular adapters for binding both the E2~ubiquitin thioester and the substrate to accomplish ubiquitin transfer directly [7,8]. For HECT-type ligases, there is a common bilobal HECT domain, which contains a larger N-lobe that interacts with E2 ligases, and a smaller C-lobe that contains the active-site cysteine [9]. The HECT E3s form a covalent bond between ubiquitin and C-lobe to form a ubiquitin-E3 thiole-ester intermediate before transferring it to the target protein [5]. The RBR or TRIAD [two RING fingers and a DRIL (double RING finger linked motif)] E3 ubiquitin ligases have common features of the larger RING and HECT ubiquitin ligases [10,11]. However, unlike single subunit RING- or HECT-style E3 ligases, RBR E3 ubiquitin ligases are multidomain proteins, containing a N terminal RING finger (RING1), an IBR (in between rings) or DRIL, and a C terminal RING finger (RING2) [12].

Ubiquitination especially involving E3 ubiquitin ligases, plays an important role in modulation of plant immunity [13,14,15]. Living in complex environment conditions, plants face a lot of abiotic and biotic stress and develop a series of adaptation mechanisms. Plants can recognize microbial- or pathogen-associated molecular patterns (MAMP/PAMP) using transmembrane pattern recognition receptors (PRRs) to trigger the PAMP/pattern-triggered immunity (PTI), which is the first line of defense response. Pathogen could secrete effectors to suppress this line of plants. Then plants would activate the effector-triggered immunity (ETI) to contrast infection [16]. More and more reports demonstrate E3 ubiquitin ligases such as the RING, U-box, HECT, and CRL types are involved in various process of plant PTI, ETI, and signal pathways [2,3,13,15]. However, the functions of RBR E3 ligases in plant immunity are still little known.

*Ralstonia solanacearum* is a soil-borne plant pathogenic bacterium, which is considered as a complex species with extensive diversity, and is widely distributed in tropical, subtropical, and some temperate regions [17]. This pathogen could cause plant bacterial wilt in more than 50 plant families, especially many important economic crops, resulting in huge economic losses around the world [17]. There are many strategies that have been developed to control bacterial wilt, including cultural practices, biological control, integrated management, etc. [18]. However, bacterial wilt has remained a challenging problem in agricultural crop protection. Cultivating resistant varieties is one of the most environmentally friendly, economical, and effective methods, which is regarded as a key approach for integrated management of bacterial wilt [18]. A deep understanding of the role of resistance-related genes will help with breeding new resistant varieties.

In this study, we analyze the function of RBR E3 ligase *NtRNF217* in *Nicotiana tabacum* responses to *R. solanacearum*. The results of this study provide evidence for the role of RBR E3 ligases in plant immunity, which may help with disease management of *R. solanacearum*.

## 2. Results

### 2.1. Amino Acids Sequence Analyses of NtRNF217

**Figure 1:**
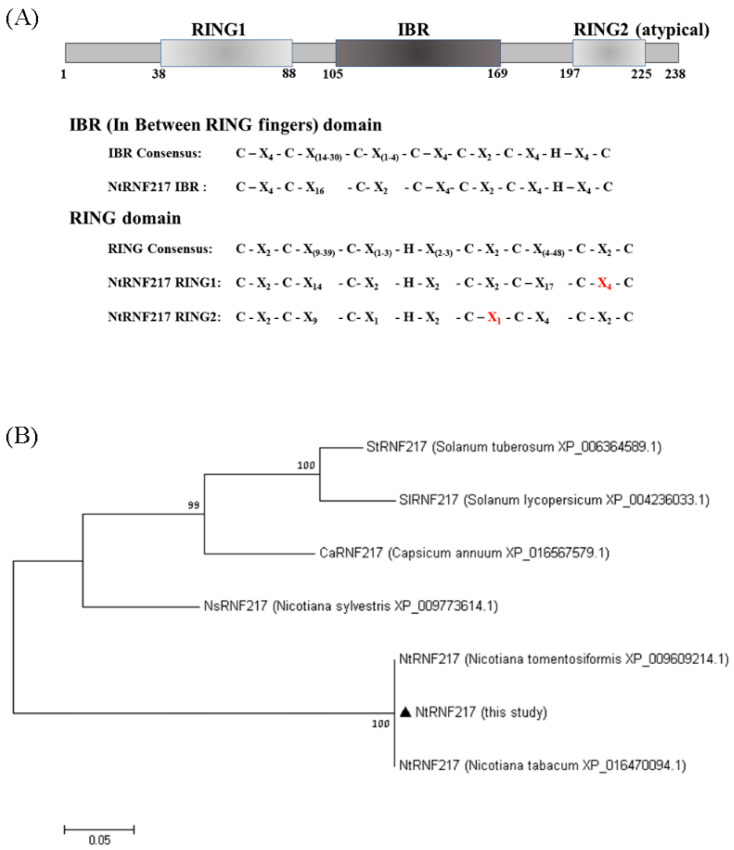
Sequence analyses of NtRNF217. (**A**) Diagram of the NtRNF217 protein with the functional domains annotated, and comparison of the domains to the consensus sequences. RING means Really Interesting New Gene; IBR means in between rings. (**B**) Phylogenetic tree of RNF217 protein sequences from different species. Values at the branches indicate the percentage of bootstrap support for 1000 resamplings.

Using PROSITE search, three domains were identified in NtRNF217: a variant N terminal RING finger (RING1), an IBR, and an atypical RING finger (RING2) at the C terminal end (Figure 1A). This means that NtRNF217 belongs to the RBR E3 ligase protein. However, compared with the consensus RBR sequence, in the RING1 of NtRNF217, there were four amino acids between Cysteins 7 and 8 rather than two consensus amino acids, and in the RING2 of NtRNF217, there was only one amino acid between Cysteins 6 and 7 rather than two consensus amino acids. Aligning NtRNF217 with other RNF217 from Solanaceae, the protein sequence of *N. tabacum* was similar to that of *N. tomentosiformis*, and distant to that of *Capsicum* and *Solanum* (Figure 1B).

### 2.2. Response of NtRNF217 Transcript Levels to R. solanacearum and Exogenous Hormones

**Figure 2:**
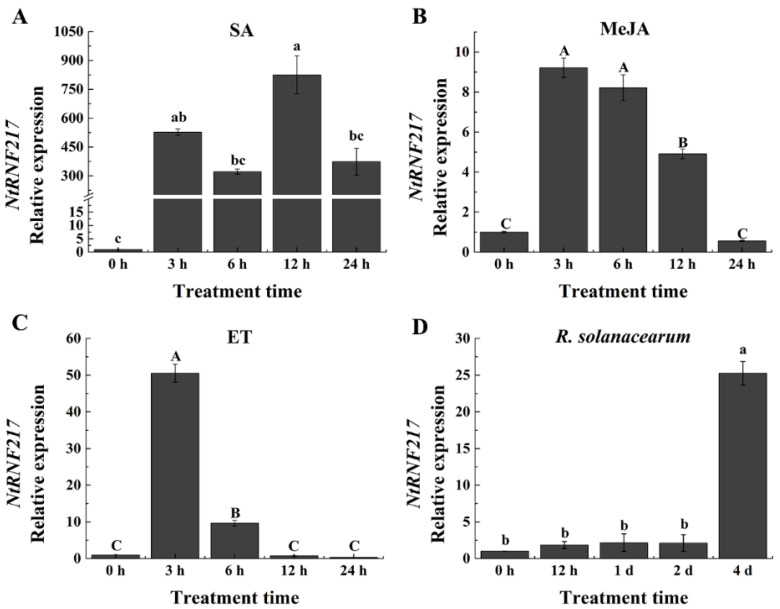
qRT-PCR analysis of the relative *NtRNF217* transcript levels in WT-Yunyan87 tobacco plants treated with exogenous hormones and pathogen. (**A**–**C**) *NtRNF217* transcript levels measured at different time points in WT-Yunyan87 tobacco after treatment with salicylic acid (SA, 2 mM), methyl jasmonate (MeJA, 0.1 mM), and ethephon (ET, 7 mM). (**D**) *NtRNF217* transcript levels measured in WT-Yunyan87 tobacco after *R. solanacearum* inoculation. Plants were inoculated by soil drenching with 10 mL cell suspension of *Ralstonia solanacearum* (10^8^ CFU mL^−1^). Error bars indicate the standard error; values were based on three independent replicates. *NtUBI3* used as the reference gene. Different letters indicated significant differences as determined by Tukey HSD (uppercase difference *p*-value < 0.01; lowercase difference *p*-value < 0.05).

Salicylic acid (SA), jasmonic acid (JA), and ethylene (ET) are important resistance signal molecules in plants. To test whether *NtRNF217* plays a role in plant resistance, qRT-PCR (quantitative real-time PCR) was used to analyze the relative expression of *NtRNF217* after hormones treatment. As shown in Figure 2A–C, the expression of *NtRNF217* in tobacco significantly fluctuated within 24 h post treatment (hpt) after ethephon, methyl jasmonate (MeJA), and SA treatment. We found transcript levels of *NtRNF217* to be increased by 50.52-fold and 9.22-fold at 3 hpt with 7 mM ethephon and 0.1 mM MeJA application, individually, before slowly returning to the original state. In response to 2 mM SA treatment, the transcript levels of *NtRNF217* were significantly enhanced, and reached the maximum at 12 hpt, almost 824.9-fold compared with the control.

The relative expression of *NtRNF217* in WT-Yunyan87 also significantly changed after *R. solanacearum* infection, reaching the peak at the fourth day after inoculation (Figure 2D). In a word, the *NtRNF217* gene is inducible by different hormones and *R. solanacearum*.

### 2.3. Overexpression of NtRNF217 Enhances Resistance of Tobacco to R. solanacearum

**Figure 3:**
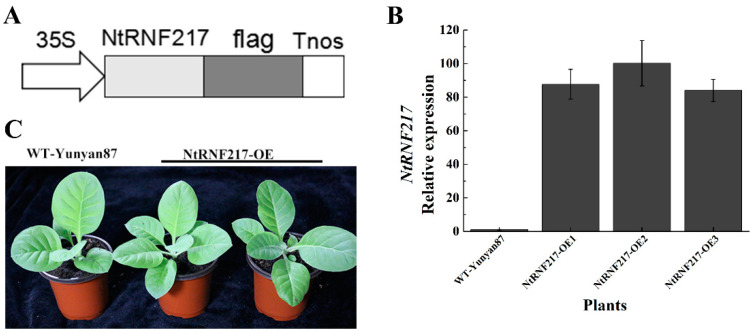
Generation of *NtRNF217*-overexpressing tobacco plants. (**A**) Schematic diagram of the 35S::NtRNF217-flag fusion protein construct. (**B**) The relative transcripts of *NtPRNF217* in transgenic plants and WT-Yunyan87 plants were tested by qRT-PCR. (**C**) Physiological phenotypes of *NtRNF217*-OE and WT-Yunyan87 plants.

The high expression levels of *NtRNF217* in response to *R. solanacearum* inoculation and hormone treatments had initially determined that the *NtRNF217* gene played a role in plant resistance response. To clarify its role in tobacco resistance to *R. solanacearum*, we generated transgenic overexpression-*NtRNF217* tobacco plants under the control of 35S promoter in the pBWAHS-ccdb vector (Figure 3A). Eventually, the cultivated overexpressed plants were analyzed focusing on the *NtRNF217* transcript levels compared with WT-Yunyan87 plants by qRT-PCR. The result showed that the transcript levels of *NtRNF217* in transgenic tobacco plants were significantly higher than for WT-Yunyan87 (Figure 3B). There were not any phenotypic differences between WT-Yunyan87 and *NtRNF217*-OE transgenic plants (Figure 3C), which were used in the next experiments.

**Figure 4:**
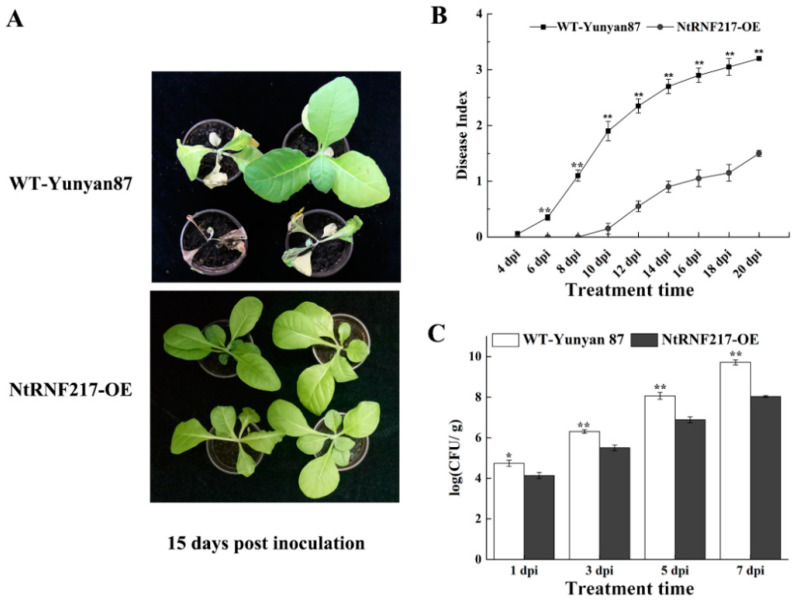
Overexpression of *NtRNF217* enhances tobacco resistance to *Ralstonia solanacearum*. (**A**) The phenotypes of representative *NtRNF217*-OE and wild-type (WT-Yunyan87) plants 15 days post inoculation with *R. solanacearum*. (**B**,**C**) Disease index of WT-Yunyan87 and *NtRNF217*-OE plants inoculated by *R. solanacearum* and the growth of *R. solanacearum* in roots. Plants were irrigated with a 10 mL suspension of the highly virulent *R. solanacearum* strain CQPS-1 (approximate 10^8^ CFU mL^−1^). ‘dpi’ means day post inoculation. Error bars indicated the standard error of three independent biological replicates, Asterisks indicated a statistically significant (*t*-test, * *p* < 0.05 or ** *p* < 0.01).

For exploring the ability of *NtRNF217*-OE plants to resist to bacterial wilt, plants were inoculated with the high virulent *R. solanacearum* strain CQPS-1 through root irrigation. At 15 days after inoculation (dpi), WT-Yunyan87 plants distinctly showed wilt symptoms, while *NtRNF217*-OE plants were in a healthy state (Figure 4A) and showed a significantly lower disease index (Figure 4B). Spread of *R. solanacearum* in the roots of *NtRNF217*-OE plants was also significantly lower than WT-Yunyan87 within 7 days after inoculation (Figure 4C). These results suggested that *NtRNF217* can promote tobacco resistance to *R. solanacearum*.

### 2.4. Overexpression of NtRNF217 Increases the Accumulation of H_2_O_2_ and O_2_^−^ Production

**Figure 5:**
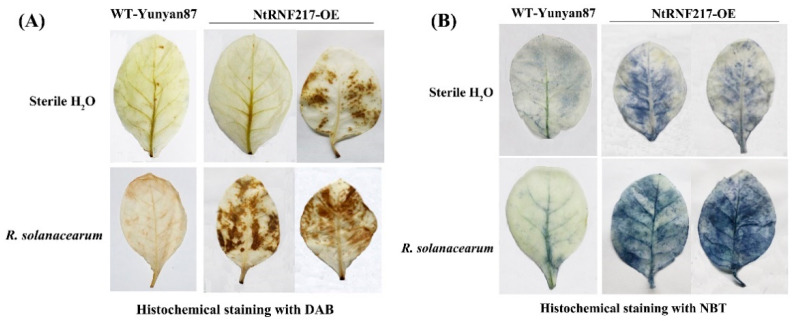
Histochemical staining. DAB (**A**) and NBT (**B**) staining of WT-Yunyan87 and *NtRNF217*-OE plants before inoculation and after 24-h *Ralstonia solanacearum* inoculation. DAB: 3,3-diaminobenzidine, and NBT: nitro blue tetrazolium.

In order to observe the local defense responses of tobacco after *R. solanacearum* infection, H_2_O_2_ and O_2_^−^ accumulation, a part of reactive oxygen species (ROS), were detected by histochemical staining. One day after inoculation by *R. solanacearum*, transgenic tobacco accumulated higher levels of H_2_O_2_ compared to WT-Yunyan87 tobacco, while there was no difference in the accumulation of H_2_O_2_ between *NtRNF217*-OE and WT-Yunyan87 plants treated with sterile water (Figure 5A). Similarly, when the bacteria were not inoculated, the production of O_2_^−^ in *NtRNF217*-OE plants was not significantly different from WT-Yunyan87 plants. After *R. solanacearum* inoculation, the accumulation of O_2_^−^ in *NtRNF217*-OE plants was significantly higher than in WT-Yunyan87 plants (Figure 5B).

### 2.5. Overexpression of NtRNF217 Enhances the Antioxidant System of Tobacco

**Figure 6:**
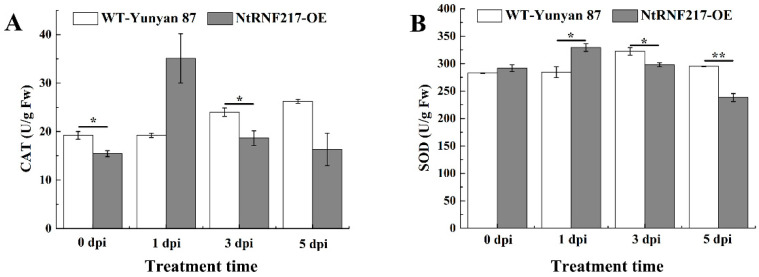
The activities of CAT and SOD in WT-Yunyan87 and *NtRNF217*-OE plants after inoculation with 10 mL *Ralstonia solanacearum* suspension (approximate 10^8^ CFU mL^−1^). (**A**) CAT activity. (**B**) SOD activity. dpi means day post inoculation. Error bars indicate the standard error of three overexpression lines. Asterisks indicate a significant difference determined by *t*-test (* *p* < 0.05 or ** *p* < 0.01).

Previous experiments have shown that overexpression of *NtRNF217* can enhance the accumulation of ROS. To further explore the effect of *NtRNF217*-overexpression on tobacco antioxidant system, the activity of catalase (CAT) and superoxide dismutase (SOD) in WT-Yunyan87 and *NtRNF217*-OE plants within 5 dpi after *R. solanacearum* inoculation were tested. As shown in Figure 6, the activities of CAT and SOD fluctuated and peaked at 1 day post inoculation in *NtRNF217*-OE plants, which is faster than in WT-Yunyan87 plants (Figure 6A,B). In general, the activities of the antioxidant enzymes in *NtRNF217*-OE plants were increased more quickly than that in wild type plants after *R. solanacearum* inoculation.

### 2.6. Overexpression of NtRNF217 Activates the Expression of Defense-Related Genes

**Figure 7:**
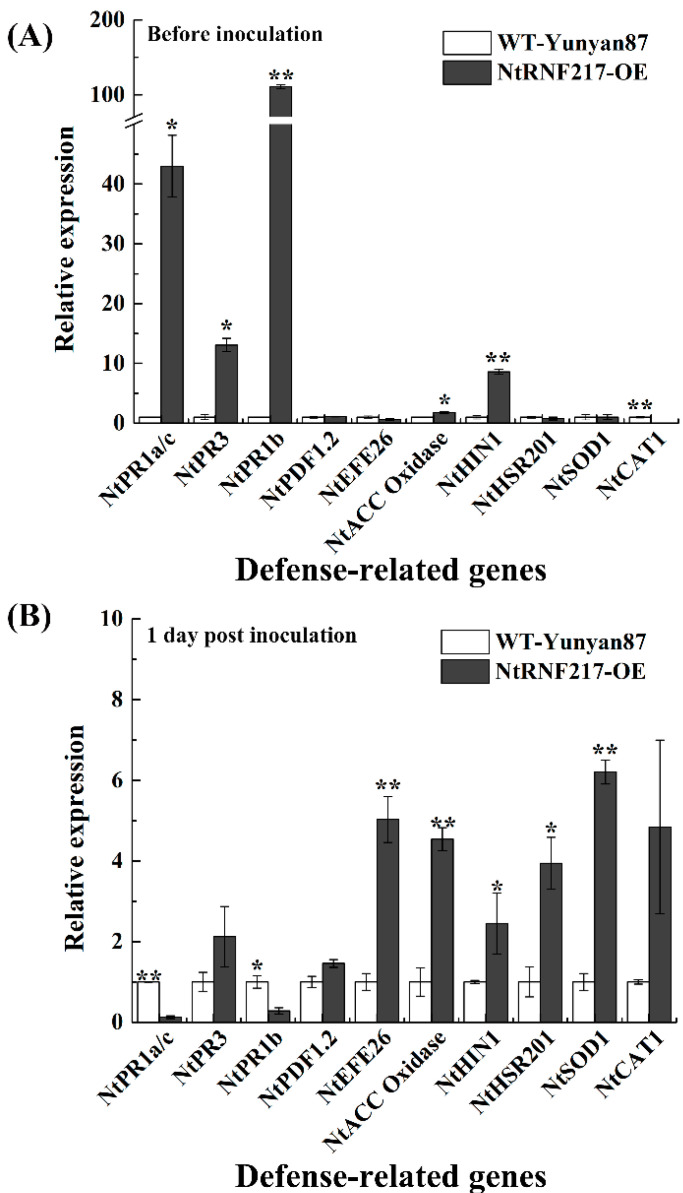
The relative gene expressions before and after inoculation with *Ralstonia solanacearum*. qRT-PCR was performed before (**A**) and 1 day post inoculation (**B**). Plants were inoculated with 10 mL of *R. solanacearum* cells (approximate 10^8^ CFU mL^−1^) by root irrigation. The *NtUBI3* gene was used as a reference gene. The gene transcript levels in WT-Yunyan87 plants were converted to 1 and used as controls. Error bars indicate the standard error of three overexpression lines. Asterisks indicate a significant difference determined by *t*-test (* *p* < 0.05 or ** *p* < 0.01).

In order to further understand the resistance mechanism, the expression of defense-related genes in non-infected and infected tobacco plants were tested (Figure 7), including the SA-responsive genes *NtPR1a/c* and *NtPR3*, JA-responsive genes *NtPR1b* and *NtPDF1.2*, ET production-associated genes such as *NtEFE26* and *NtACC Oxidase*, HR-associated genes *NtHIN1* and *NtHSR201*, and ROS detoxification-associated genes *NtCAT1* and *NtSOD1* [19,20,21,22,23,24]. Before *R. solanacearum* infection, the transcript levels of *NtPR1a/c*, *NtPR3*, *NtPR1b*, *NtACC Oxidase*, and *NtHIN1* were significantly higher in *NtRNF217*-OE plants compared with that in WT-Yunyan87 plants, increasing 42.97-fold, 13.06-fold, 110.81-fold, 1.77-fold, and 8.61-fold, respectively (Figure 7A). The expression levels of *NtEFE26*, *NtACC Oxidase*, *NtHIN1*, *NtHSR201*, and *NtSOD1* were significantly higher in *NtRNF217*-OE than WT-Yunyan87 plants 24 h post inoculation, increasing 5.03-fold, 4.54-fold, 2.45-fold, 3.95-fold, and 6.21-fold, respectively (Figure 7B).

## 3. Discussion

E3 ubiquitin ligases have a large number of members, which mediate transportation of ubiquitin from an E2 Ub-conjugating enzyme to the target substrate, to confer specificity to ubiquitination [1]. Previous reports have shown that E3 ubiquitin ligases, such as maize RING-finger protein *ZmRFP1* Gene and Pepper E3 Ubiquitin Ligase RING1 Gene *CaRING1* are involved in plant immunity and regulate the adaptability of organisms to biotic and abiotic stress [25,26]. RBR E3 ligases are recently identified members of E3 ubiquitin ligases, containing two RINGs linked by an IBR domain [11]. They are important in the regulation of human and animal immune signaling, but there is still little known about their role in plant immunity [15,27,28]. In this study, we demonstrated that the RBR-type E3 ligase *NtRNF217* in *N. tabacum* participated in immune regulation against *R. solanacearum* infection.

There is evidence that E3 ubiquitin ligases is important for control both of production of the HR and restriction of pathogen growth [29]. As a soil-borne plant pathogenic bacterium, *R. solanacearum* is able to enter root tissues from soil, and then invade the plant vascular system and cause the wilt of plants. The wilting symptoms causing by *R. solanacearum* are associated with strong bacterial multiplication in xylem vessels and abundant production of exopolysaccharides (EPS) [30]. The results of this study showed that overexpression of *NtRNF217* in *N. tabacum* significantly reduced multiplication of *R. solanacearum* and inhibited the development of disease symptoms after irrigating pathogen compared with wild-type plants.

Histochemical staining demonstrated that *NtRNF217*-OE tobacco plants rapidly accumulated H_2_O_2_ and O_2_^−^ after 24 h of *R. solanacearum* infection (Figure 5). H_2_O_2_ and O_2_^−^ are part of ROS, which are usually considered as toxic by-products in the normal metabolism of plants and play important role in the defense response of plants [31,32,33]. On the one hand, ROS can trigger cell death at plant-infected sites, leading to hypersensitive response (HR) [34,35]. On the other hand, ROS may also act as a second messenger to regulate the expression of disease resistance-related genes and initiate transcription of plant antitoxin synthesis genes [36]. However, excessive accumulation of ROS would cause damage to the plant cells, so there is an antioxidant system in plants responsible for holding the steady state, which contains SOD, CAT, APX (ascorbate peroxidase), etc. [37,38]. Previous research has verified that E3 ubiquitin ligase can enhance the resistance of tobacco to abiotic stress by regulating the antioxidant system [26]. In the present study, the activities of SOD and CAT in *NtRNF217*-OE tobacco were increased at 1 day after inoculation (Figure 6). What is more, the relative expressions of *NtCAT1* and *NtSOD1* in *NtRNF217*-OE plants were significantly higher than in WT-Yunyan87 plants 24 h after inoculation with *R. solanacearum* (Figure 7). It could be speculated that *NtRNF217*-OE tobacco plants rapidly produce a large number of ROS when infected by *R. solanacearum*.

SA, JA, and ET are important signaling molecules in the plants, playing significant roles in regulating plant defense responses against different stress [39]. The expression levels of *NtRNF217* were induced by exogenous SA, JA, and ET, indicating that *NtRNF217* is involved in plant resistance (Figure 2). Overexpression of *NtRNF217* in *N. tabacum* significantly improved the transcript levels of SA-responsive genes *NtPR1a/c* and *NtPR3*, JA-responsive genes *NtPR1b* without *R. solanacearum* infection (Figure 7), suggesting that *NtRNF217* involved in regulating signal pathways. A previous study has reported that E3 ubiquitin ligase CUL3^BPM^ regulated the JA signal pathway through targeted JA-pathway regulators MYC2, MYC3, and MYC4 proteins [40]. As an E3 ubiquitin ligase, the target of *NtRNF217* is still unknown, which should be further studied. Solving this problem is the key to understanding how *NtRNF217* gene participates in the regulation of signal pathways.

Localized HR and plant-wide systemic acquired resistance (SAR) are two well-characterized defense strategy in vascular plants [41]. SAR is a defense response that is activated at the infected site and triggered to the distal part to protect undamaged tissues [39,42]. It is known that SAR is a SA-dependent response, and is accompanied by the expression of various pathogenesis-related (PR) genes [42]. The qRT-PCR analysis showed that the NtRNF217 overexpression enhanced the expression of large range of defense-related genes, including the SA-responsive genes (Figure 7A). These results seem to support the conclusion that *NtRNF217* is involved in plant SAR. Warding off diverse biotroph pathogens in plants, SAR confers broad-spectrum immunity [43]. It could be speculated that *NtRNF217* may play an important role in resisting other biotic or abiotic stresses, which requires further verification.

Although a series of proteins related to *R. solanacearum* resistance have been reported in different plants, such as the WRKY family of pepper, PR family proteins of tobacco, etc. [44,45], there was no specific resistance gene found until now. This is also the biggest problem for breeding resistant varieties to control bacterial wilt. More research and methods should be used to analyze the functions of various resistance-related genes and regulatory networks, which would provide new ideas for future genetic breeding.

## 4. Materials and Methods

### 4.1. Plant Materials

A tobacco (*N.* *tabacum*) cultivar Yunyan 87, provided by Yuxi Zhong Yan Tobacco Seed CO., LTD, Yuxi, China, was used as a wild type (WT-Yunyan87) and for the transformation experiments in this study. The sterile plants to be used for obtaining transgenic plants were prepared following previous report [45].

### 4.2. Characterization of the NtRNF217 Gene and Construction of Over-Expressing Plants

Sequence of the *NtRNF217* gene was assessed by BLAST and protein domains were identified using PROSITE pattern search. Alignment with reference sequences from NCBI was done using Clustal W [46]. Phylogenetic analysis was performed using neighbor-joining (NJ) and the algorithm of Poisson model with 1000 bootstrap resamplings in MEGA version 7 [47]. The GenBank accession number of NtRNF217 sequence is MK578824.

The full-length cDNA of *NtRNF217* and flag sequences were amplified by RNF217 and flag primers (Table S1, see Supplementary Materials), respectively. The two sequences were inserted into Eco31I restriction sites of the binary vector pBWAHS-ccdb by digestion and ligation. The positive recombinant plasmid, pBWAHS-NtRNF217-flag, was transformed into *Agrobacterium tumefaciens* strain EHA 105, and then was transformed into WT-Yunyan87 using the leaf discs method [48]. Transgenic tobaccos were selected using hygromycin (25 mg L^−1^) and further confirmed by measuring the relative expression of *NtRNF217*. Three overexpression lines were screened for follow-up experiments. The overexpressed plants used for this study were obtained by asexual reproduction on MS medium [49], and then transferred to peat substrate. Greenhouse conditions were controlled at 25 ± 2 °C with 16/8 h light/dark cycle and a relative humidity of 80%.

### 4.3. Application of Plant Hormones and Exogenous Inducers

WT-Yunyan87 plants at the four-leaf stage were sprayed with three plant hormones as previously described [45]: MeJA (0.1 mM), ethephon (7 mM), and SA (2 mM). The treated leaves were harvested at the indicated timepoints—0, 3, 6, 12, and 24 hpt, then were frozen and stored at −80 °C for the qRT-PCR analysis. The experiment was repeated three times.

### 4.4. Pathogens and Inoculation Procedures

*R. solanacearum* strain CQPS-1 (phylotype I/biovar 3), as highly virulent pathogen in this study [50], was cultured in B medium [51]. Soil drenching was used for testing pathogenicity, monitoring bacterial growth assay in roots, detecting the expression of resistance-related genes, and evaluating the activities of CAT and SOD: plants with four to five true leaves were inoculated with 10 mL inoculum (OD_600_ = 0.1, approximate 10^8^ CFU mL^−1^ cell suspension). The pathogenicity tests were made with 10 plants and were repeated three times. The incidence and disease index were monitored every day for 20 days using a 0 to 4 scale as described by Dang et al. [52]. The leaves were harvested before or 24 h post inoculation for preparation of RNA. The activities of CAT and SOD were determined after 1 day, 3 days, and 5 days post inoculation by collecting the leaf samples. When collecting leaf samples, consisting of mixed leaves from two plants. At indicated time points 1, 3, 5, and 7 days post inoculation, the root samples were harvested for counting the number of colonies [45].

Infiltrated inoculation was used to study H_2_O_2_ and O_2_^−^ accumulation in response to *R. solanacearum* infections. Plants were inoculated by bacterial suspension into the second leaves from the top using a syringe without a needle, and leaf samples were harvested at 0 h and 24 h.

### 4.5. Histochemical Staining and SOD, CAT Activities

H_2_O_2_ and O_2_**^−^** accumulation were detected by histochemical staining with 3,3-diaminobenzidine (DAB) and nitro blue tetrazolium (NBT), respectively [53,54]. After 24 h of infiltrating inoculum (5 × 10^7^ CFU mL^−1^) into the second leaves from the bottom using a syringe without a needle, the leaves of WT-Yunyan87 and transgenic plants were dyed with 100 µg mL^−1^ DAB or NBT solution. Stained leaf samples were boiled in 97% (*v*/*v*) ethanol for removing chlorophyll, then photographed directly using a camera (Canon, EW-78E, Tokyo, Japan). SOD activity was determined by a NBT light reduction method described by Qu et al. [55]. CAT was assayed by following the methods described by Qin et al. [56].

### 4.6. RNA Extraction, cDNA Synthesis, and Quantitative Real-Time PCR

The mixed leaves samples of two plants from different treatment (approximate 0.1 g) were frozen in liquid nitrogen and ground into powder using mortars. Total RNA was extracted by TRNzol reagent (TIANGEN, Beijing, China). Then RNA samples were reverse transcribed with iScript™ cDNA Synthesis Kit (BIO-RAD, Hercules, CA, USA). qRT-PCR was used to analyze the relative transcript levels of various genes according to the previous description [45]; primers are shown in Table S1 (see Supplementary Materials).

## 5. Conclusions

In summary, our study provides evidence that *NtRNF217* acts as a positive regulator in response to *R. solanacearum* infection, depending on production of a large number of ROS, regulating an antioxidant system and defense-related genes. As a member of RBR-type E3 ligases, *NtRNF217* certainly participates in ubiquitination process; however, its target protein is still unknown. Further research should be carried out to elucidate this question.

## Data Availability

The datasets used and/or analyzed during the current study are available from the corresponding author on reasonable request.

## Supplementary Materials

The following are available online at https://www.mdpi.com/article/10.3390/ijms22115507/s1.
